# DSC, FTIR and Raman Spectroscopy Coupled with Multivariate Analysis in a Study of Co-Crystals of Pharmaceutical Interest

**DOI:** 10.3390/molecules23092136

**Published:** 2018-08-24

**Authors:** Patrycja Garbacz, Marek Wesolowski

**Affiliations:** Department of Analytical Chemistry, Medical University of Gdansk, Gen. J. Hallera 107, 80416 Gdansk, Poland; patrycja89103@gumed.edu.pl

**Keywords:** DSC, FTIR, Raman spectroscopy, PCA, CA, indomethacin, saccharin, furosemide, *p*-aminobenzoic acid

## Abstract

Co-crystals have garnered increasing interest in recent years as a beneficial approach to improving the solubility of poorly water soluble active pharmaceutical ingredients (APIs). However, their preparation is a challenge that requires a simple approach towards co-crystal detection. The objective of this work was, therefore, to verify to what extent a multivariate statistical approach such as principal component analysis (PCA) and cluster analysis (CA) can be used as a supporting tool for detecting co-crystal formation. As model samples, physical mixtures and co-crystals of indomethacin with saccharin and furosemide with *p*-aminobenzoic acid were prepared at API/co-former molar ratios 1:1, 2:1 and 1:2. Data acquired from DSC curves and FTIR and Raman spectroscopies were used for CA and PCA calculations. The results obtained revealed that the application of physical mixtures as reference samples allows a deeper insight into co-crystallization than is possible with the use of API and co-former or API and co-former with physical mixtures. Thus, multivariate matrix for PCA and CA calculations consisting of physical mixtures and potential co-crystals could be considered as the most profitable and reliable way to reflect changes in samples after co-crystallization. Moreover, complementary interpretation of results obtained using DSC, FTIR and Raman techniques is most beneficial.

## 1. Introduction

To reach their target area of the human body, orally administered active pharmaceutical ingredients (APIs) must dissolve in gastrointestinal fluids and then pass through cell membranes. The first stage, dissolution, is vital to the entire process. The poor water solubility of APIs (defined as a solubility of less than 100 µg/mL), constitutes one of the most important limiting factors in a drug’s development [1], and is the most frequent cause for APIs not being used in the clinic, despite possessing the desired therapeutic properties. Even the toxicity of APIs and insufficient medicinal activity in the clinical trial stage are much rarer reasons for the discontinuation of trials compared to insufficient aqueous solubility. Difficulties at all stages of drug development mean that less than 1% of potential APIs have a chance to reach the market as drugs [2]. The problem of poor water solubility affects approximately 40% of oral APIs with immediate-release used in modern pharmacotherapy.

For this reason, attention has recently been paid to co-crystals—a new form of APIs, which influence their bioavailability, mainly water solubility, dissolution rate or chemical and physical stability. The definition of co-crystal was set by the Food and Drug Administration (USA) in 2013 [3]. According to this guidance, co-crystals consist of at least two ingredients in the solid state, connected via non-covalent bonding in the same crystal lattice. One ingredient of co-crystals is an API, the second a neutral component, the so-called co-former.

Several methods have been used to study co-crystals, such as thermal, spectroscopic and X-ray diffraction techniques. Based on the change of melting point of ingredients in comparison with their physical mixture, differential scanning calorimetry (DSC) allows the quick detection of co-crystal formation [4]. Thermogravimetry (TG) enables differentiation between solvation and co-crystallization of API [4,5]. Infrared with Fourier transformation (FTIR) [6,7], near infrared (NIR) [8] and Raman [9,10] spectroscopies, in turn, are commonly used as tools allowing recognition of structural modification in co-crystal lattice by non-covalent bonding formation. Furthermore, NIR can be used on-line as a method for monitoring co-crystal formation [11,12]. DSC coupled with Raman microscopy or fibre-optic Raman analyzers has also been applied for real-time monitoring of the co-crystallization process [10,13], while powder X-ray diffraction and single-crystal X-ray diffraction tools provide deep insights into co-crystal structures [14,15].

It is worth noting that reliable data can only be obtained through the use of several methods—it is impossible to confirm co-crystal formation using one method exclusively. Nevertheless, during interpretation of the data acquired using the above methods, many problems could have arisen, hence, the necessity of searching for additional tools to facilitate interpretation of the data and allowing further conclusions to be drawn. Recently, the chemometric approach has attracted the greatest interest as a supporting tool confirming co-crystal formation. Among multivariate methods, principal component analysis (PCA) has been the focus of the greatest attention. Based on the data acquired from NIR spectra, PCA was applied to visualize co-crystallization carried out using the slow evaporation method [11,12]. PCA allows the understanding of the key stages in the process and subsequently could contribute to improved control of parameters during co-crystallization. NIR spectroscopy coupled with PCA was also useful as a method for observation of co-crystallization pathways, when processes were carried out using different approaches of the same method of co-crystal formation [16,17]. Moreover, PCA applied on the data acquired from Raman spectroscopy was also a valuable tool in expanding the knowledge of co-crystal formation by the grinding method [18].

In the light of all the above, the main concept behind the work was to verify to what extent a multivariate statistical approach such as PCA and cluster analysis (CA) can be used as a supporting tool for detecting co-crystal formation. This study will constitute one contribution towards enhancing our knowledge of relations between co-crystals, physical mixtures, APIs and co-formers based on their thermal and spectral properties, ultimately leading towards the development of a simple and reliable tool for the detection of co-crystallization between pharmaceutical ingredients.

## 2. Results and Discussion

To realize the objective of the study, widely investigated co-crystals of indomethacin with saccharin [8,10,16,18,19,20,21] and furosemide with *p*-aminobenzoic acid [12,14,22] were chosen as the model samples. Their crystal structure is already known to consist of ingredient dimers connected via hydrogen bonds. Further, DSC and FTIR and Raman spectroscopies were used to study indomethacin and furosemide co-crystals and the subsequent data were used for PCA and CA calculations.

### 2.1. Co-Crystals of Indomethacin with Saccharin

**Figure 1:**
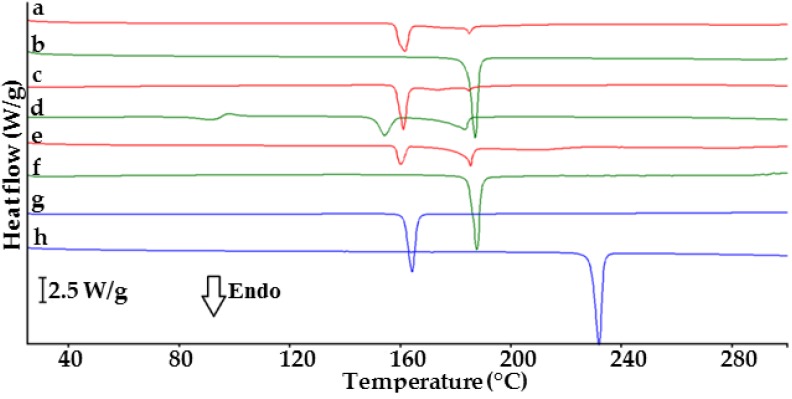
DSC curves of indomethacin-saccharin physical mixtures at API/co-former ratios: (a) 1:1, (c) 2:1, (e) 1:2; indomethacin-saccharin co-crystals at API/co-former ratios: (b) 1:1, (d) 2:1, (f) 1:2; (g) indomethacin, (h) saccharin.

DSC curves of API, co-former, their physical mixtures and co-crystals are presented in Figure 1. Indomethacin (curve g) and saccharin (curve h) display single endothermic peaks due to melting at 161.9 and 229.1 °C, respectively [18,23]. Their mixtures (curves a, c and e) show two endothermic peaks at temperatures lower than the melting points of ingredients. The first peak appears at ~159 °C for all physical mixtures, probably due to the melting of the eutectic mixture [20]. The heats of fusion differ for each mixture. The second peak at ~184 °C confirms the melting of co-crystals. Co-crystal at 1:1 molar ratio (curve b) shows an endothermic peak at 185.3 °C, in line with the literature data [18]. A single endothermic peak at 186.1 °C (curve f) implies co-crystallization at 1:2 molar ratio. Co-crystal at 2:1 molar ratio (curve d) shows three endothermic peaks at 90.9, 153.7 and 182.9 °C and exothermic at 97.8 °C, which excludes co-crystal formation.

To confirm these findings and to gain a more profound insight into phase changes in physical mixtures and potential co-crystals when heated, CA and PCA methods were applied. Three matrices were constructed for the data acquired from the DSC curves. Matrix I consisted of the data for API, co-former and potential co-crystals, matrix II included the data for physical mixtures and potential co-crystals, while matrix III was formed by joining the data included in the two previous matrices, i.e., data for API, co-former, physical mixtures and potential co-crystals.

**Figure 2:**
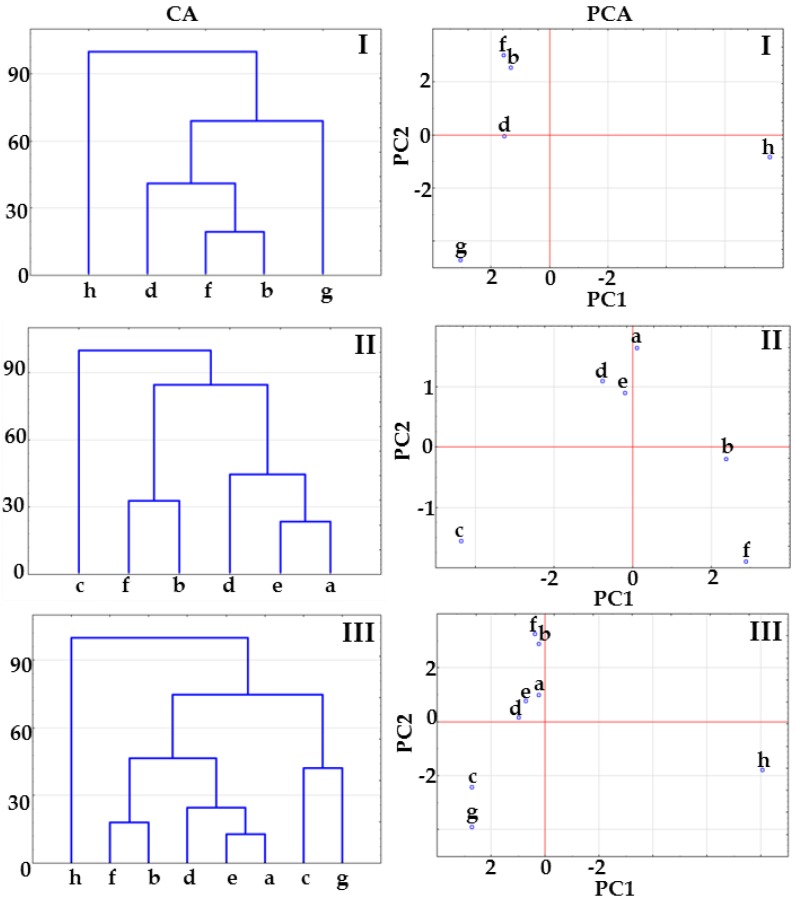
CA tree diagrams and PCA score scatter plots for matrices I, II and III obtained using the data acquired form DSC curves. Indomethacin-saccharin physical mixtures at API/co-former ratios: (a) 1:1, (c) 2:1, (e) 1:2; indomethacin-saccharin co-crystals at API/co-former ratios: (b) 1:1, (d) 2:1, (f) 1:2; (g) indomethacin, (h) saccharin.

The results of CA calculations presented in Figure 2 show that co-crystals at 1:1 and 1:2 molar ratios create a cluster below 33% of maximum distance. The co-crystal at 2:1 molar ratio joins to the co-crystal cluster above 33% of maximum distance (Figure 2, CA, matrix I) or links to the physical mixture cluster above 33% of maximum distance (matrix II, Figure 2, CA) or below 33% of maximum distance (Figure 2, CA, matrix III). This arrangement of samples on CA tree diagrams could reveal co-crystal formation at 1:1 and 1:2 molar ratios, while excluding the possibility of co-crystallization at 2:1 molar ratio.

PCA score scatter plots for the same matrices are illustrated in Figure 2. All the plots revealed that co-crystals at 1:1 and 1:2 molar ratios are located in the same plane revealing the great similarity between samples, regardless of the fact that in the case of matrix II, co-crystals are differentiated by the PC2 axis including ~20% of the total variance (Table 1). Co-crystal at 2:1 molar ratio is located on the same plane along the PC1 and PC2 axes with physical mixtures at 1:1 and 1:2 molar ratios (Figure 2, PCA, matrices II and III), which suggest a similarity to physical mixtures. Thus, CA and PCA data imply that co-crystals at 1:1 and 1:2 molar ratios are formed, while co-crystallization at 2:1 molar ratio did not occur.

**Figure 3:**
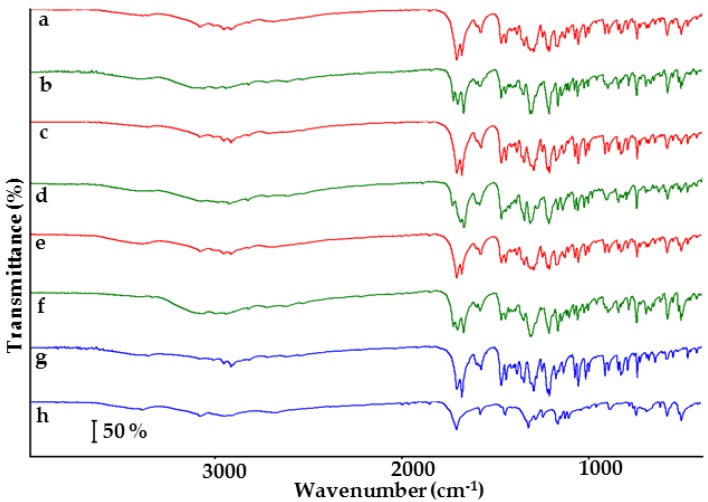
FTIR spectra of indomethacin-saccharin physical mixtures at API/co-former ratios: (a) 1:1, (c) 2:1, (e) 1:2; indomethacin-saccharin co-crystals at API/co-former ratios: (b) 1:1, (d) 2:1, (f) 1:2; (g) indomethacin, (h) saccharin.

FTIR spectra of the samples under study are shown in Figure 3. Characteristic bands of indomethacin (spectrum g) are found at 3965.3 and 2926.6 cm^−1^ (O–H stretching vibration), 1717.0 cm^−1^ (C=O stretching of carboxylic acid dimer), 1691.5 cm^−1^ (C=O stretching of benzoyl group), 1307.7 cm^−1^ (C–O), and 1068.0 cm^−1^ (C–Cl). The saccharin (spectrum h) shows characteristic bands at 3093.5 cm^−1^ (N–H stretching), 2973.5 cm^−1^ (C–H stretching), 2695.0 cm^−1^ (O–H stretching), 1720.6 cm^−1^ (C=O stretching), 1136.0 cm^−1^ (SO_2_ asymmetric stretching), and 1177.5 cm^−1^ (symmetric stretching). These data are compatible with those found in the literature [22,24]. Physical mixtures (spectra a, c and e) display bands of both ingredients, suggesting that co-crystallization does not occur. Nevertheless, mixtures differ from each other in band transmittance. FTIR spectrum of co-crystal at 1:1 molar ratio (spectrum b) differs markedly from that of physical mixture at the same molar ratio. Co-crystal spectrum reveals new bands at 3084.1, 3006.7 and 1736.5 cm^−1^, whereas the characteristic bands for physical mixture at 3092.9, 2965.5 and 2926.6 cm^−1^ disappeared. The band at 1691 cm^−1^ (physical mixture) was shifted to 1681 cm^−1^ in the co-crystal spectrum. These differences are consistent with the literature data and confirm the formation of hydrogen bonding during co-crystallization [25]. The co-crystal spectrum at 1:2 molar ratio (spectrum f) also differs from that of physical mixture at the same molar ratio. The characteristic bands for co-crystal at 1:1 molar ratio are also found in the 1:2 co-crystal spectrum with insignificant changes in spectral position.

**Figure 4:**
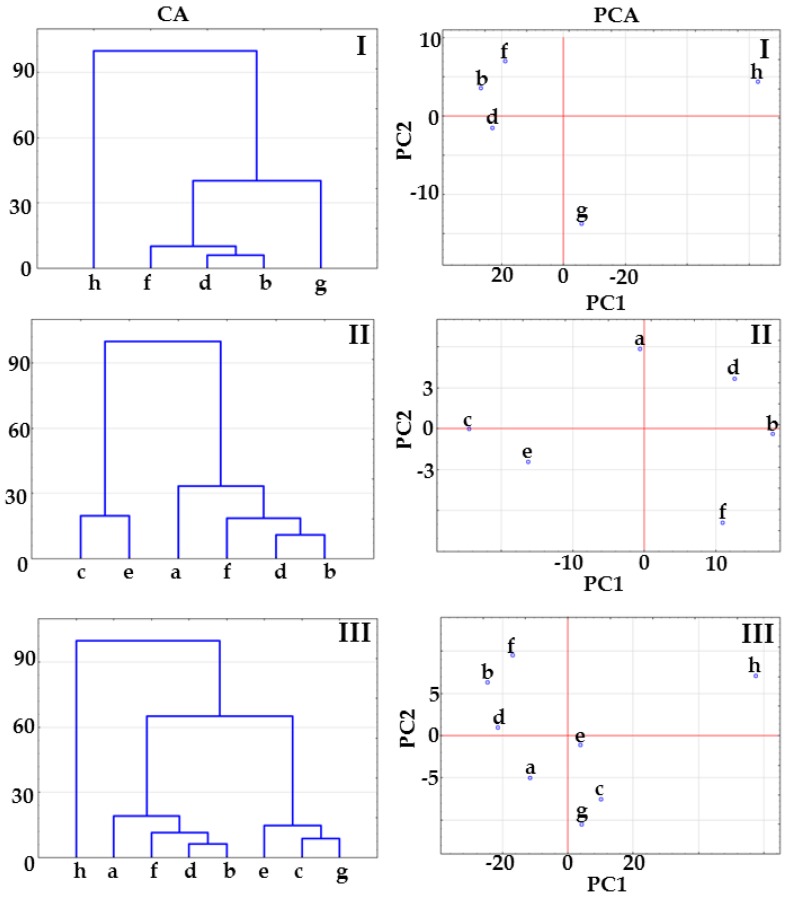
CA tree diagrams and PCA score scatter plots for matrices I, II and III obtained using the data acquired form FTIR spectra. Indomethacin-saccharin physical mixtures at API/co-former ratios: (a) 1:1, (c) 2:1, (e) 1:2; indomethacin-saccharin co-crystals at API/co-former ratios: (b) 1:1, (d) 2:1, (f) 1:2; (g) indomethacin, (h) saccharin.

The shifting of FTIR bands is also observed in the spectrum of co-crystal at 2:1 molar ratio (spectrum d), but this does not occur at the same spectral position as in the spectrum of co-crystal at 1:1 molar ratio, i.e., bands for 1:1 molar ratio co-crystal are found at 1736.5, 1712.8 and 1681.9 cm^−1^, whereas those for 2:1 molar ratio co-crystal are displayed at 1740.7, 1698.1 and 1678.6 cm^−1^. The results of CA calculations are shown in Figure 4. For all the matrices, tree diagrams revealed that co-crystals at different molar ratios are grouped in a separate cluster below 33% of maximum distance. In the case of matrices II and III, co-crystals create a cluster with physical mixture at 1:1 molar ratio below 33% of maximum distance. This implies that co-crystallization occurs regardless of the molar ratio of ingredients, but also creates uncertainty over the interpretation of results.

A detailed inspection of the data compiled in Table 1 revealed that PC1 explains over 90% of the total variance for all FTIR matrices. Thus, PC1 is a key factor discriminating the samples under study, whereas PC2 and subsequent PCs do not contribute to discrimination because total variance is too low. The PCA score scatter plot for matrix I illustrated in Figure 4 shows that co-crystals are distinctly isolated from indomethacin and saccharin along the PC1 axis. A similar situation can be observed for matrix II, with co-crystals being clearly separated from physical mixtures. Figure 4 (PCA) for matrix III confirms the findings for matrices I and II. Co-crystals are located on the left side of the plot, physical mixtures and API in the central section, with co-former on the right. It is worth noting that for all matrices, co-crystal at 1:1 molar ratio is slightly distinguished from other co-crystals along the PC1 axis.

Loading factors for the first three principal components (PC1, PC2 and PC3) calculated applied the FTIR and Raman data are compiled in Table 2. Detailed inspection of these data reveals that spectral ranges 1717–1679 cm^−1^, 1240–1182 cm^−1^ and 1363–1305 cm^−1^ are the most important in the first loading for all the matrices. Spectral zone between 1717 and 1679 cm^−1^ reflects carbonyl stretching vibrations of indomethacin and saccharin. In this region characteristic shift of bands due to co-crystals formation are also observed. Moreover, matrix II is influenced by loading ~1737 cm^−1^ assigned to co-crystallization. This suggests that changes in spectral bands due to co-crystals formation probably significantly influences arrangement of samples on PCA. 

**Figure 5:**
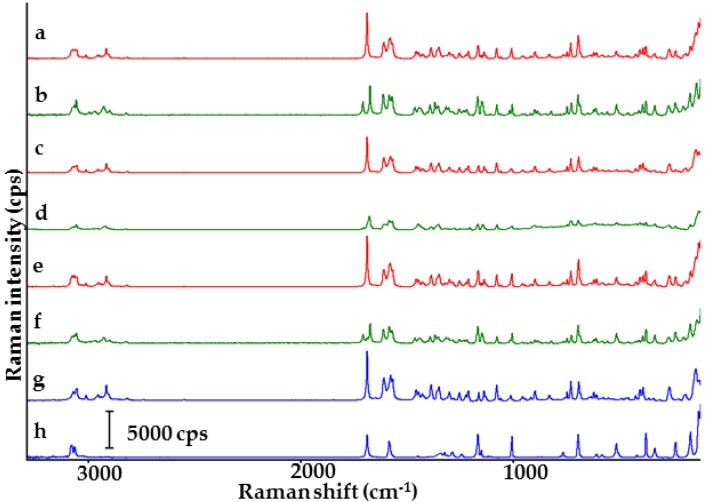
Raman spectra of indomethacin-saccharin physical mixtures at API/co-former ratios: (a) 1:1, (c) 2:1, (e) 1:2; indomethacin-saccharin co-crystals at API/co-former ratios: (b) 1:1, (d) 2:1, (f) 1:2; (g) indomethacin, (h) saccharin.

Score scatter plot Raman spectra of the samples are shown in Figure 5. Indomethacin (spectrum g) displays sharp bands at 1697.0 cm^−1^ (C=O benzoyl stretching vibration), 1617.8 cm^−1^ (C=C stretching) and 1586.9 cm^−1^ (ring vibration of indole). Saccharin shows intensive bands at 1697.0 cm^−1^ (C=O stretching vibration), 1014.7 cm^−1^ (SO_2_ stretching vibration) and 1593.0 cm^−1^ (C–C stretching vibration) [10]. Physical mixtures of both ingredients (spectra a, c and e) reveal characteristic bands for API and co-former with no significant changes in their spectral position, confirming the lack of interaction between ingredients. Spectra of co-crystals (spectra b, d and f) differ from those of physical mixtures. The band at 1697 cm^−1^ in the spectra of physical mixtures is replaced by two new bands at ~1716 and ~1682 cm^−1^ in the co-crystal spectra. Further, the spectral position of Raman bands of co-crystals at 1:1 and 1:2 molar ratios are practically identical, differing solely in intensity. Besides this, in the spectra of co-crystals at 1:1 and 2:1 molar ratios slight differences are detected in spectral position. For instance, the co-crystal at 1:1 molar ratio shows a band at 1682.8 cm^−1^, while its equivalent at 2:1 molar ratio reveals the same band at 1686.4 cm^−1^.

**Figure 6:**
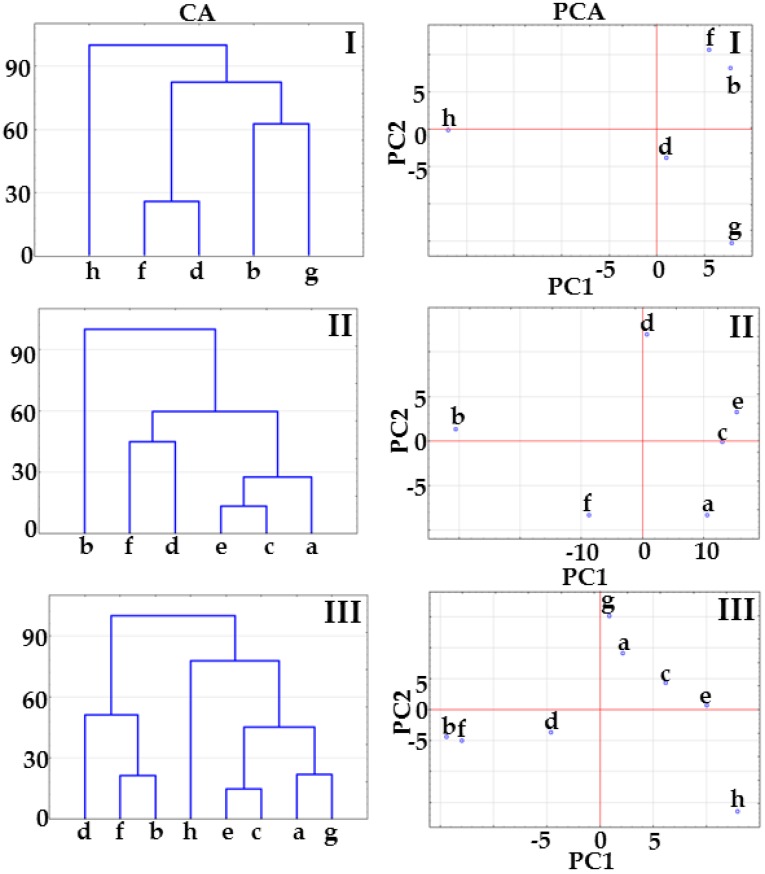
CA tree diagrams and PCA score plots for matrices I, II and III obtained using the data acquired from Raman spectroscopy. Indomethacin-saccharin physical mixtures at API/co-former ratios: (a) 1:1, (c) 2:1, (e) 1:2; indomethacin-saccharin co-crystals at API/co-former ratios: (b) 1:1, (d) 2:1, (f) 1:2; (g) indomethacin, (h) saccharin.

CA and PCA plots reflect the differences in spectral positions and Raman intensities of the samples in question (Figure 6). Tree diagrams for matrices I and III show that co-crystals at 1:1 and 1:2 molar ratios create a cluster below 33% of maximum distance due to the great similarity between samples. The tree diagram for matrix II allows us to draw a negative conclusion. Co-crystal at 1:1 molar ratio links to the remaining samples at 100% of maximum distance (completely dissimilar), while co-crystals at 2:1 and 1:2 molar ratios link together at ~45% of maximum distance. Physical mixtures are gathered into a separate cluster below 33% of maximum distance.

The data compiled in Table 1 reveal that the most reliable results in PCA calculations were obtained for matrix II. PC1 and PC2 gathered together explain more than 95% of total variance in the data set, in contrast to 80% of total variance for matrices I and III. Thus, the score scatter plot for matrix II enables the clear separation of co-crystal at molar ratio 1:1 from the remaining samples, i.e., co-crystals at molar ratios 2:1 and 1:2 and all the physical mixtures.

Loading factors summarized in Table 2 for Raman data show that spectral zones at 1703–1695 cm^−1^ and 1683–1680 cm^−1^ are the most significant in the first loading for matrices II and III and for arrangement of samples on PCA plots. The former spectral range is related to intensive bands, which reflect C=O stretching vibrations of indomethacin and saccharin. The latter, in turn, is assigned to co-crystals. In the case of matrix I, the most important in the PC1 loading is region between 1622 and 1614 cm^−1^, related to band characteristic for indomethacin.

Taking all the above into consideration, the results of CA and PCA calculations using the data acquired from DSC curves exclude co-crystal formation at 2:1 molar ratio because this sample forms a cluster together with physical mixtures. Co-crystallization could only occur at 1:1 and 1:2 molar ratio. The distribution of samples on CA tree diagrams and PCA score scatter plots based on the FTIR and Raman data suggests co-crystallization regardless of the molar ratio of ingredients. However, it should be emphasized that co-crystal at 1:1 molar ratio is slightly isolated from the remaining co-crystals along the PC1 axis in the case of PCA plots based on FTIR data (PC1 explained more than 90% of total variance, Table 1). What is more, on the PCA plot based on the Raman data (Figure 4, PCA, matrix II) the separation along PC1 between co-crystal at 1:1 molar ratio and the remaining samples is significant. This arrangement of samples confirms co-crystallization at 1:1 molar ratio, but does not exclude co-crystallization at 2:1 and 1:2 molar ratios. In brief, the implication from the CA and PCA study is that co-crystal was formed at 1:1 molar ratio, co-crystallization at 2:1 molar ratio did not occur, while co-crystals at 1:2 molar ratio cannot be excluded. Thus, further study is required to confirm this hypothesis.

### 2.2. Co-Crystals of Furosemide with p-Aminobenzoic Acid

**Figure 7:**
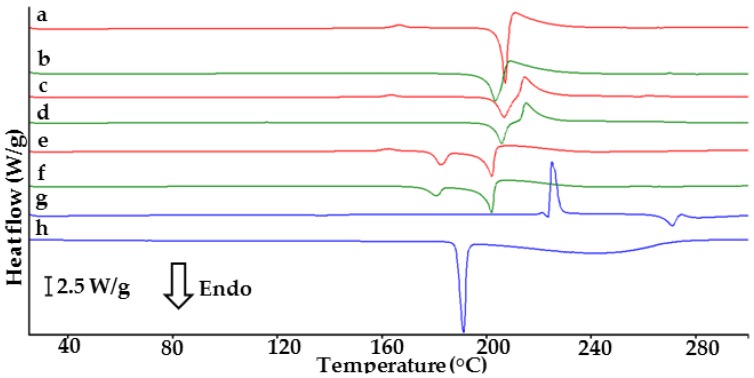
DSC curves of furosemide-*p*-aminobenzoic acid physical mixtures at API/co-former ratios: (a) 1:1, (c) 2:1, (e) 1:2; furosemide-*p*-aminobenzoic acid co-crystals at API/co-former ratios: (b) 1:1, (d) 2:1, (f) 1:2; (g) furosemide, (h) *p*-aminobenzoic acid.

Figure 7 illustrates the DSC curves of furosemide, *p*-aminobenzoic acid, physical mixtures of both ingredients and co-crystals. API (curve g) shows two endothermic and one exothermic peak. The first of these, due to polymorphic transition, appears at ~137 °C, the second at 226.2 °C followed by an exothermic is probably due to melting with decomposition of furosemide [24,26]. The DSC curve of co-former (curve h) also displays two endothermic peaks, the first sharp at 188.7 °C due to melting and the second broad peak at 244.6 °C reflects probable evaporation of the melted substance [27,28].

Physical mixtures of API and co-former (Figure 7, curves a, c and e) display a peak at 136 °C assigned to polymorphic transition of furosemide and an exothermic event at ~165 °C. Furthermore, endothermic followed by exothermic peaks are observed for physical mixtures at 1:1 and 2:1 molar ratios. In the case of physical mixture at 1:2 molar ratio (curve e), apart from an exothermic peak at 157.8 °C and an endothermic at 201.2 °C, the presence of an additional endothermic peak at 181.8 °C is probably due to the melting of *p*-aminobenzoic acid. Co-crystals at 1:1 and 2:1 molar ratios (curves b and d) display single endothermic peaks at 202.5 and 204.95 °C, respectively, followed by an exothermic peak. However, on the DSC curve of co-crystal at 2:1 molar ratio (curve d), a slight endothermic peak appears at ~137 °C probably indicating the presence of furosemide in unbound form. Similarly to physical mixture at 1:2 molar ratio, co-crystal at 1:2 molar ratio (curve f) reveal an additional endothermic peak at 180.3 °C. Thus, DSC allows us to conclude that co-crystal is formed at a 1:1 molar ratio. This result is consistent with the literature data [14,22].

**Figure 8:**
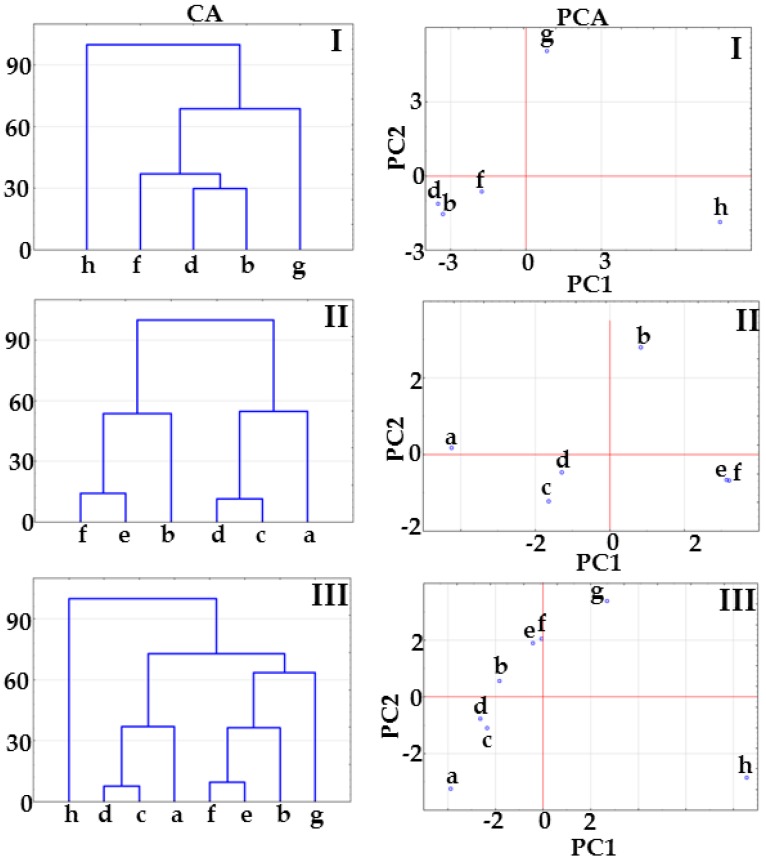
CA tree diagrams and PCA score scatter plots for matrices I, II and III obtained using the data acquired form DSC curves. Furosemide-*p*-aminobenzoic acid physical mixtures at API/co-former ratios: (a) 1:1, (c) 2:1, (e) 1:2; furosemide-*p*-aminobenzoic acid co-crystals at API/co-former ratios: (b) 1:1, (d) 2:1, (f) 1:2; (g) indomethacin, (h) saccharin.

Results of CA calculations for matrix I presented in Figure 8 reveal that co-crystals form a cluster below 33% of maximum distance. This suggests co-crystallization whatever the ingredient molar ratios. Tree diagrams for matrices II and III show that potential co-crystals at 2:1 and 1:2 molar ratios are grouped in a cluster with their physical mixtures. This excludes co-crystallization at 2:1 and 1:2 molar ratios. Co-crystal at 1:1 molar ratio is separate from the remaining samples. This arrangement of samples supports the supposition that co-crystal is obtained only at a 1:1 molar ratio.

Results of PCA calculations for matrix I (Figure 8) also reveal that API and co-former are distinctly separate from potential co-crystals at 1:1, 2:1 and 1:2 molar ratios, which are grouped on the same plane along the PC1 and PC2 axes. Unfortunately, no reliable conclusions can be drawn as to whether or not co-crystallization actually occurred at various molar ratios. PCA score scatter plots for matrices II and III do confirm that co-crystals are not formed at 2:1 and 1:2 molar ratios, since these samples are grouped with their physical mixtures on the same plane along the PC1 and PC2 axes. Moreover, 1:1 molar ratio co-crystal is distinctly separate from the remaining samples, which confirms co-crystallization. Thus, it is most likely that co-crystal at 1:1 molar ratio was obtained, while co-crystallization at 2:1 and 1:2 molar ratios has not been confirmed.

**Figure 9:**
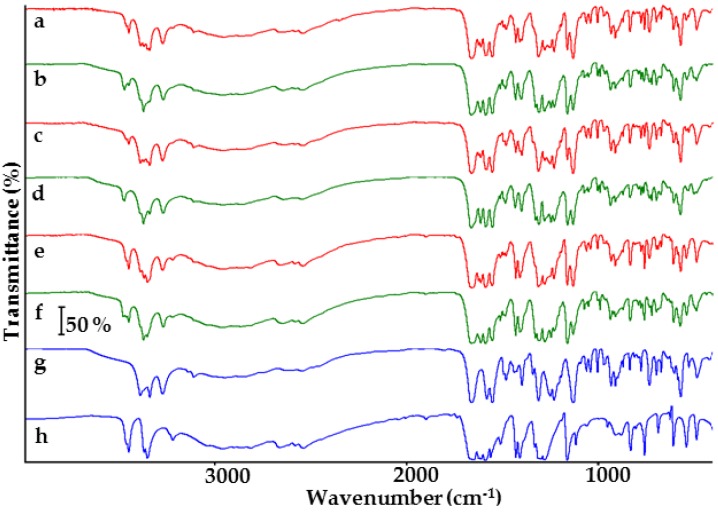
FTIR spectra of furosemide-*p*-aminobenzoic acid physical mixtures at API/co-former ratios: (a) 1:1, (c) 2:1, (e) 1:2; furosemide-*p*-aminobenzoic acid co-crystals at API/co-former ratios: (b) 1:1, (d) 2:1, (f) 1:2; (g) furosemide, (h) *p*-aminobenzoic acid.

Figure 9 illustrates the FTIR spectra of the samples under study. Characteristic bands for furosemide (spectrum g) are found at 3399.4 and 3350.3 cm^−1^ (NH_2_ stretching vibration), 3284.2 cm^−1^ (N–H stretching), 1672.0 cm^−1^ (C=O stretching), 1591.8 cm^−1^ (N–H bending), 1322.5 cm^−1^ (R–SO_2_ symmetric stretching), 1442.1 cm^−1^ (S–O stretching) and 744.6 cm^−1^ (C–Cl stretching). *p*-Aminobenzoic acid (spectrum h) shows characteristic bands at 3460.4 and 3363.5 cm^−1^ (NH_2_ stretching), 1662.8 cm^−1^ (C=O stretching), 1624.3 cm^−1^ (NH_2_ in plane deformation), 1600.8 cm^−1^ (NH_2_ scissoring vibration), 1573.5 cm^−1^ (COO stretching) and 1422.1 cm^−1^ (COO stretching). These data comply with those found in the literature [22,29,30]. Spectra of physical mixtures (spectra a, c and e) display bands characteristic to both ingredients, with only a slight shift of bands observed. Significant difference in intensity of transmittance is probably due to the different molar ratios of ingredients. In contrast to physical mixtures, the band assigned to the amino group of *p*-aminobenzoic acid shifts from ~3460 cm^−1^ to ~3482 cm^−1^ in spectra of co-crystals at 1:1 and 2:1 molar ratios (spectra b and d). In addition, bands attributed to the amino group of furosemide (3399.4 and 3350.3 cm^−1^) disappear in the co-crystal spectra. Alterations in the spectral position of bands are connected with hydrogen bonding formation and confirm that co-crystals can indeed be obtained. Spectrum f (co-crystal at 1:2 molar ratio) does not differ from that of physical mixture at the same molar ratio, but changes in bands transmittance intensity are observed.

**Figure 10:**
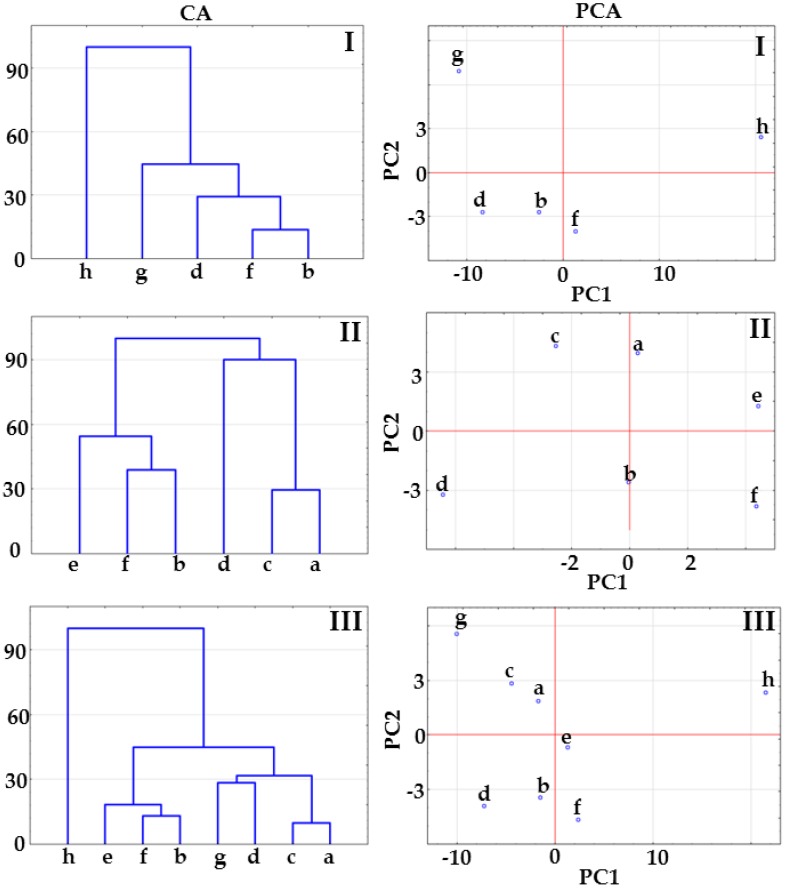
CA tree diagrams and PCA score scatter plots for matrices I, II and III obtained using the data acquired form FTIR spectra. Furosemide-*p*-aminobenzoic acid physical mixtures at API/co-former ratios: (a) 1:1, (c) 2:1, (e) 1:2; furosemide-*p*-aminobenzoic acid co-crystals at API/co-former ratios: (b) 1:1, (d) 2:1, (f) 1:2; (g) indomethacin, (h) saccharin.

The CA tree diagram for matrix I, consisting of the data acquired from the FTIR spectra presented in Figure 10, shows that potential co-crystals are linked together below 33% of maximum distance and differ significantly from both ingredients. Figure 10 (CA, matrices II and III) illustrates to what extent co-crystals are similar to the physical mixtures under study. Figure 10 (CA, matrix II) shows that co-crystals at 1:1 and 1:2 molar ratios create a cluster with physical mixture at 1:2 molar ratio, whereas physical mixtures at 1:1 and 2:1 molar ratios are gathered together below 30% of maximum distance. This cluster is joined with the co-crystal at 2:1 molar ratio. The distribution of samples is completely different when furosemide and *p*-aminobenzoic acid are included into matrix III. The cluster composed of co-crystals at 1:1 and 1:2 molar ratios is linked with physical mixture at 1:2 molar ratio below 20% of maximum distance, while co-crystal at 2:1 molar ratio is grouped with furosemide below 33% of maximum distance. This introduces significant uncertainty into data interpretation.

The PCA score scatter plots presented in Figure 10 show that potential co-crystals are grouped in a lower part of the plot in such a way that co-crystal at 1:1 molar ratio is located between co-crystals at 1:2 and 2:1 molar ratios. The co-crystal at 1:2 molar ratio is separated from *p*-aminobenzoic acid along the PC1 and PC2 axes, while the co-crystal at 2:1 molar ratio is separated from furosemide along the PC2 axis. All the co-crystals are separated from physical mixtures along PC2 with ~40% (PCA, matrix II) and ~12% (PCA, matrix III) of total variance (Table 1). The data obtained using CA and PCA suggest co-crystal formation irrespective of molar composition. Co-crystals are distinctly separated from physical mixtures in CA and PCA plots for matrix II. What is more, co-crystal at 2:1 molar ratio is separated from both co-crystals and physical mixtures.

Loading factors specified in Table 2 for FTIR data show that the most important spectral zones for all matrices in the first loading are spectral ranges 773–769 cm^−1^, 1425–1421 cm^−1^ and band around 3460 cm^−1^. These spectral areas reflect the characteristic bands for furosemide and *p*-aminobenzoic acid in the samples studied. Regarding the second loading, the main spectral ranges are following—1343–1340 cm^−1^ and 1178–1166 cm^−1^.

**Figure 11:**
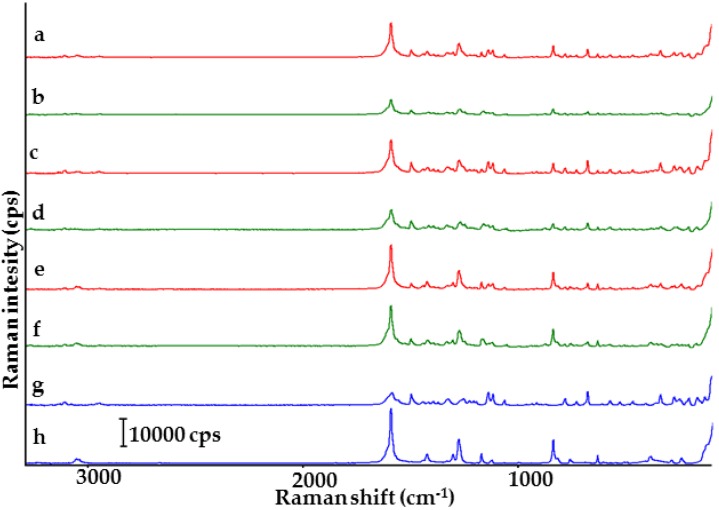
Raman spectra of furosemide-*p*-aminobenzoic acid physical mixtures at API/co-former ratios: (a) 1:1, (c) 2:1, (e) 1:2; furosemide-*p*-aminobenzoic acid co-crystals at API/co-former ratios: (b) 1:1, (d) 2:1, (f) 1:2; (g) furosemide, (h) *p*-aminobenzoic acid.

Figure 11 presents Raman spectra for the samples. Characteristic bands of furosemide (spectrum g) occur at 1593.5 cm^−1^ (NH_2_ scissoring vibration), 1503.1 cm^−1^ (aromatic ring stretching), 1334.2 cm^−1^ (SO_2_ asymmetric stretching), 1144.6 cm^−1^ (SO_2_ symmetric stretching) and 681.8 cm^−1^ (C–Cl stretching). Characteristic bands for *p*-aminobenzoic acid (spectrum h) are found at 1599.4 cm^−1^ (NH_2_ scissoring vibration) and 1283.9 cm^−1^ (C–OH stretching vibration) [29,30]. The spectra of physical mixtures confirm characteristic bands for both ingredients. The differences in intensity of Raman bands are probably due to the varying molar composition of the mixtures. A comparison of the spectra of co-crystals with those of physical mixtures reveals slight differences in the spectral position of bands. Thus, for instance, the band assigned to the hydroxyl group of *p*-aminobenzoic acid is shifted from ~1283 cm^−1^ in the spectra of physical mixtures to ~1279 cm^−1^ in the co-crystal spectra at 1:1 and 2:1 molar ratios, while in the co-crystal spectrum at 1:2 molar ratio the above band is shifted to 1282 cm^−1^. Furthermore, bands at ~1178 and ~1147 cm^−1^ in the physical mixture spectra are replaced by a band at ~1170 cm^−1^ in the co-crystal spectra at 1:1 and 1:2 molar ratios, while the co-crystal spectrum at 2:1 molar ratio show bands at 1169 and 1147 cm^−1^. Co-crystals also differ in the Raman intensity of bands in each of the spectra.

**Figure 12:**
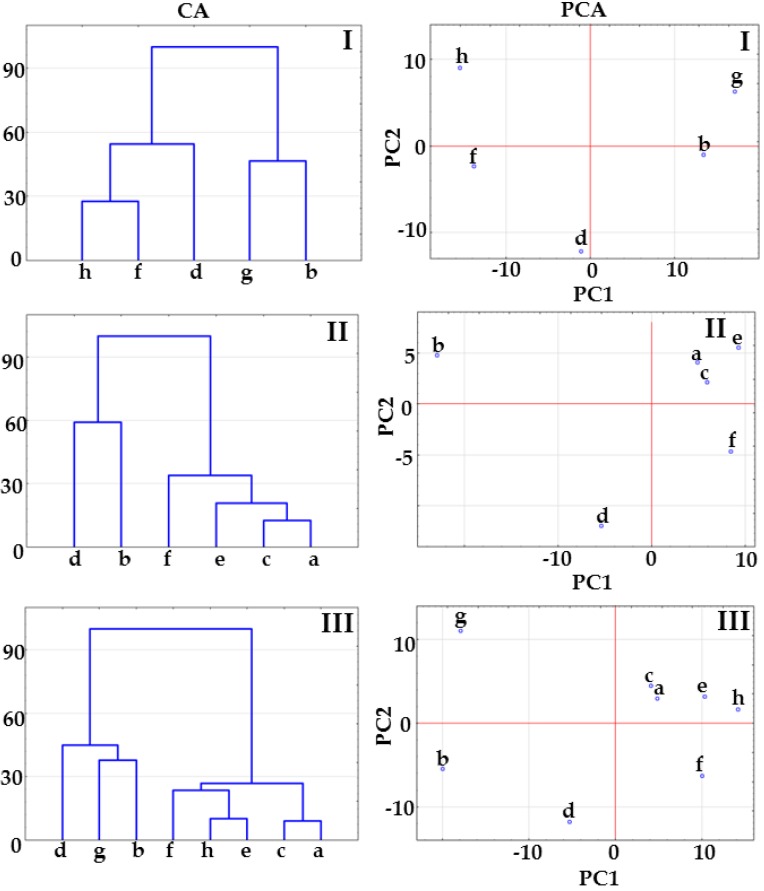
CA tree diagrams and PCA score scatter plots for matrices I, II and III obtained using the data acquired form Raman spectroscopy. Furosemide-*p*-aminobenzoic acid physical mixtures at API/co-former ratios: (a) 1:1, (c) 2:1, (e) 1:2; furosemide-*p*-aminobenzoic acid co-crystals at API/co-former ratios: (b) 1:1, (d) 2:1, (f) 1:2; (g) indomethacin, (h) saccharin.

The CA tree diagrams based on the Raman spectra are shown in Figure 12. In the case of matrix I, co-crystals at 1:1 and 2:1 molar ratios create separate clusters above 33% of maximum distance, demonstrating that co-crystals differ both from each other and from the remaining samples. The co-crystal at 1:2 molar ratio is grouped with physical mixtures (matrix II) or *p*-aminobenzoic acid (matrix III). This suggests that co-crystallization did not occur.

The PCA score scatter plots illustrated in Figure 12 confirm the findings derived from the CA calculations. The co-crystal at 1:1 molar ratio is clearly separated from the physical mixtures and remaining co-crystals along the PC1 axis and from furosemide along the PC2 axis (Figure 10, PCA, matrices II and III). The co-crystal at 1:2 molar ratio creates a cluster with *p*-aminobenzoic acid (matrix I) and *p*-aminobenzoic acid and physical mixtures (matrix III). In the case of all matrices, the co-crystal at 2:1 molar ratio is located separately in the middle section of the score scatter plots, and PC1 discriminates this co-crystal from all the remaining samples—ingredients, co-crystals and physical mixtures.

Loading factors for Raman data (Table 2) indicates that all the matrices in the first loading are to the greatest extent influences by spectral ranges 1614–1595 cm^−1^ and around 843 cm^−1^, both due to intensive bands in *p*-aminobenzoic acid spectrum. Important in the first loading is also spectral zone between 1286 cm^−1^ and 1278 cm^−1^, which reflects shift of bands related to co-crystal formation.

Taking all the above into consideration, the results of CA and PCA calculations based on the data acquired from DSC curves suggest that only co-crystal at 1:1 molar ratio is formed. Co-crystals at 2:1 and 1:2 molar ratios are grouped on the same plane along the PC1 and PC2 axes or link with their physical mixtures, which excludes co-crystallization. An inspection of the CA tree diagram and PCA score scatter plot obtained from FTIR spectra revealed co-crystallization regardless of molar composition, yet the characteristics of the samples is dissimilar. In particular, attention was paid to the co-crystal at 2:1 molar ratio, which differs from both co-crystals and physical mixtures. A CA and PCA study based on the data acquired from Raman spectroscopy confirms the results from FTIR data. The co-crystal at 1:1 molar ratio is separated from the remaining samples along PC1 axis, while the potential co-crystal at 2:1 molar ratio is separated from the remaining samples along PC1 and PC2 axes. Potential co-crystal at 1:2 molar ratio forms a cluster with physical mixtures and/or co-former, which again excludes co-crystallization. In general, co-crystals at 1:1 molar ratio were formed, while co-crystallization at 1:2 and 2:1 molar ratios did not occur. DSC data excludes co-crystallization at 2:1 molar ratio, whereas FTIR and Raman spectra encourage further study on the precise nature of the changes observed during sample grinding at 2:1 molar composition.

## 3. Materials and Methods

### 3.1. Reagents

Indomethacin, furosemide and *p*-aminobenzoic acid were purchased from Sigma Aldrich (St. Louis, MO, USA). Saccharin was acquired from Acros Organics (Morris, NJ, USA). The solvents were obtained from POCH (Polish Chemical Reagents, Gliwice, Poland). The purity of substances was ≥98%, the purity of solvent ≥99.8%.

### 3.2. Preparation of Mixtures and Co-Crystals

Binary physical mixtures of indomethacin with saccharin and furosemide with *p*-aminobenzoic acid at 1:1, 2:1 and 1:2 molar ratios were obtained by gently mixing API with co-former in an agate mortar for 5 min. Co-crystals of indomethacin with saccharin were obtained using the evaporation method. API with co-former at 1:1, 2:1 and 1:2 molar ratios were placed in a round-bottomed flask, after which 6–7 mL of ethyl acetate was added and the sample heated until it dissolved. The solutions were then left to evaporate slowly at room temperature. Co-crystals of furosemide with *p*-aminobenzoic acid were prepared using the liquid-assisted grinding method. Both ingredients were mixed at 1:1, 2:1 and 2:1 molar ratios in an agate mortar for 20 min using a pestle after the addition of 5–6 drops of acetone.

### 3.3. DSC Conditions

The DSC curves of APIs, co-formers, physical mixtures and potential co-crystals were obtained using a heat-flux DSC instrument, model 822^e^ (Mettler Toledo, Schwerzenbach, Switzerland), coupled with STARe software (version 15.00, Mettler Toledo, Schwerzenbach, Switzerland). Approximately 4 mg of sample was placed into 40 μL flat-bottomed aluminum pans which were then sealed with lids. Measurements were carried out over the range of 25 to 300 °C at a heating rate of 10 °C/min under nitrogen stream at a flux rate of 70 mL/min.

### 3.4. FTIR Conditions

A Nicolet 380 FTIR spectrometer (Thermo Fischer Scientific, Madison, WI, USA) with a DTGS KBr detector and OMNIC software (version 15.00, Mettler Toledo, Schwerzenbach, Switzerland) was employed to collect the IR spectra of the samples and a hydraulic press (Specac, Orpington, UK) to prepare pellets for FTIR analysis. Each of the pellets contained 1 mg of sample and 100 mg of KBr (Merck, Darmstadt, Germany). Measurements were carried out over the spectral range of 4000–400 cm^−1^ with resolution of 4 cm^−1^, at ambient temperature. Before each measurement, background spectra were taken with an average of 16 curves.

### 3.5. Raman Conditions

Raman spectra were recorded on a DXR Smart Raman spectrometer (Thermo Fisher Scientific, Madison, WI, USA), equipped with a 15-mW DXR 780 nm laser with a slit width of 25 µm, Raleigh filter, CCD detector and OMNIC software. The measurements were run over the range of 3413–99 cm^−1^ with resolution of 2 cm^−1^, at ambient temperature. Exposure time was 1 s (twice).

### 3.6. Multivariate Analysis

DSC curves and FTIR and Raman spectra were recorded in triplicate. Data acquired from the DSC curves, FTIR spectra and Raman spectroscopy for APIs, co-formers as well as physical mixtures and potential co-crystals at 1:1, 2:1 and 1:2 molar ratios were used as input matrices for CA and PCA calculations. Heat flow values were determined from DSC curves every 3 °C within a range of 20 and 300 °C, transmittance values from FTIR spectra and Raman intensity values collected every 3.86 cm^−1^ at a spectral range from 403.07 to 3997.91 cm^−1^ and from 303.23 to 3300.61 cm^−1^ were used as variables. The matrices obtained consisted of 98, 933 and 778 variables, for DSC, FTIR and Raman data respectively. Before CA and PCA calculations, the data acquired from Raman and FTIR spectroscopies were pre-processed with a Standard Normal Variate (SNV) algorithm [17,31,32]. SNV correction was carried out using the following equation:

(1) $$x_{corr}=\frac{x_{org}-a_{0}}{a_{1}}$$

where *x_corr_* is the corrected value, *x_org_* is the original value, *a*_0_ is the average value of the sample spectrum to be corrected, and *a*_1_ is the standard deviation of the sample spectrum.

Three matrices were prepared for each kind of measurement, DSC, FTIR and Raman, totaling 9 matrices. The first of these consisted of data for API, co-former and potential co-crystals (matrix I), the second included data for physical mixtures and potential co-crystals (matrix II), while the last matrix was formed by a combination of the data included in the two previous matrices, i.e., of data for API, co-former, physical mixtures and potential co-crystals (matrix III). Thus, the matrices consisted of, respectively 5, 6 and 8 objects for the first, second and third matrix.

PCA and CA calculations were carried out using Statistica 12 software (StatSoft Inc., Tulsa, OK, USA). For CA calculations, the Euclidean distance between pairs of objects was used and Ward’s linkage criterion according to which objects or clusters of objects are merged. A graphic visualization of the objects’ classification was presented as a tree diagram. In the case of PCA, covariance matrices were used as a basis for calculation of principal components (PCs), which means that the data were centered. The results of PCA calculations were visualized in score scatter plots of the first two PCs. PC1 and PC2 gathered together explain more than 80% of total variance.

## 4. Conclusions

The results of a CA and PCA study on all the matrices show significant variance in terms of scattering of samples, which often results in misleading conclusions about co-crystallization. The tree diagrams and score scatter plots determined for matrix I reflect the separation of co-crystals from API and co-former, which erroneously suggests co-crystal formation regardless of molar ratios. This arrangement of samples is actually due to the significant thermal (DSC) and spectral (FTIR, Raman) differences between co-crystals and output ingredients. In the case of matrix III, co-crystals are also separated from API and co-former, but are often located in proximity to physical mixtures, forming a common cluster. This has led to the incorrect classification of co-crystals as physical mixtures. This situation may well be related to the greater thermal and spectral similarity between physical mixtures and co-crystals, as opposed to physical mixtures and output ingredients. Most frequently, CA and PCA lead to satisfactory discrimination between co-crystals and physical mixtures in the case of matrix II, which contains physical mixtures and co-crystals at the same molar ratios. Both multivariate techniques enable discrimination of samples while taking account of the subtle differences in thermal and spectral profiles. It can be concluded, therefore, that application of physical mixtures as reference samples allows deeper insight into co-crystallization than the use of API and co-former or API and co-former with physical mixtures. The inference is that matrix II consisting of physical mixtures and potential co-crystals at the same molar ratios could be considered as the most profitable and reliable way to reflect the changes in samples after co-crystallization.

It is also confirmed by the fact that the most important spectral ranges in the first loading are those related to the most characteristic bands assigned to API and co-former. For this reason, the arrangement of samples on the PCA plot is a result significant spectral (FTIR, Raman) differences between co-crystals and output ingredients.

The results of CA and PCA calculations also differ with regard to the instrumental technique used for sample investigation and the multivariate method used for calculations. This derives from the fact that thermoanalytical and spectroscopic methods measure other features of the sample under study—DSC registers heat flow due to phase transitions occurring in a sample when heated. Spectroscopic methods, in turn, provide data about the chemical structure of a sample and intermolecular interactions. Thus, CA tree diagrams and PCA scores plots calculated using data acquired from FTIR and Raman spectra seem to be more reliable in the context of co-crystal detection than those derived from DSC curves. In contrast to this, CA and PCA plots based on FTIR and Raman spectra are more complex in interpretation than those obtained from DSC curves, which may result from the fact that spectroscopic methods provide more data for calculations than DSC. For this reason, complementary interpretation of the results obtained from DSC, FTIR and Raman methods can be the most beneficial and can permit co-crystal confirmation, provided that the results obtained by CA and PCA using data from DSC, FTIR and Raman are compatible.

## Figures and Tables

**Table 1:** Eigenvalues and variances in the data acquired form the DSC curves as well as FTIR and Raman spectroscopies for the ingredients, physical mixtures and co-crystals under study. Cumulative variances are given in parenthesis.

Principal Component		Indomethacin and Saccharin			Furosemide and *p*-Aminobenzoic Acid		
		Matrix I	Matrix II	Matrix III	Matrix I	Matrix II	Matrix III
**Calculation for DSC Data**							
PC1	Eigenvalue	18.23	6.70	11.58	21.80	8.59	15.88
	Variance (%)	59.04	63.90	50.04	62.94	67.49	54.78
PC2	Eigenvalue	9.64	2.14	6.40	8.25	2.10	5.70
	Variance (%)	31.20 (90.24)	20.41 (84.31)	27.65 (77.69)	23.81 (86.76)	16.47 (83.96)	19.66 (74.44)
PC3	Eigenvalue	2.46	1.12	2.62	2.77	1.77	4.57
	Variance (%)	7.97 (98.21)	10.67 (94.98)	11.30 (88.99)	8.01 (94.77)	13.87 (97.83)	15.77 (90.21)
**Calculation for FTIR Data**							
PC1	Eigenvalue	1396.59	293.44	707.85	154.68	17.30	92.34
	Variance (%)	95.00	90.42	90.91	85.63	51.93	79.14
PC2	Eigenvalue	68.03	20.14	53.41	21.10	13.44	13.84
	Variance (%)	4.63 (99.63)	6.21 (96.63)	6.86 (97.77)	11.68 (97.31)	40.36 (92.29)	11.87 (91.06)
PC3	Eigenvalue	4.77	8.77	13.27	4.28	1.66	6.21
	Variance (%)	0.32 (99.95)	2.70 (99.33)	1.70 (99.47)	2.37 (99.68)	4.97 (97.26)	5.32 (96.38)
**Calculation for Raman Data**							
PC1	Eigenvalue	157.99	305.50	101.11	226.53	153.49	171.22
	Variance (%)	48.52	80.41	41.47	65.47	71.19	65.69
PC2	Eigenvalue	106.56	58.91	94.05	68.41	47.80	53.35
	Variance (%)	32.73 (81.25)	15.51 (95.92)	38.58 (80.05)	19.77 (85.24)	22.17 (93.36)	20.47 (86.16)
PC3	Eigenvalue	58.68	9.56	41.39	50.25	13.53	34.98
	Variance (%)	18.02 (99.27)	2.52 (98.43)	16.98 (97.02)	14.52 (99.76)	6.28 (99.64)	13.42 (99.58)

**Table 2:** Loading factors for the first three principal components (PC1, PC2 and PC3) calculated on the FTIR and Raman data for the ingredients, physical mixtures and co-crystals under study.

Principal Component		Indomethacin and Saccharin			Furosemide and *p*-Aminobenzoic Acid		
		Matrix I	Matrix II	Matrix III	Matrix I	Matrix II	Matrix III
**Calculation for FTIR Data**							
PC1	Wavenumber (cm^−1^)	1240–1216 1363–1305 1717–1679	1683–1679 1328–1317 1224, 1737	1232–1220 1683–1679 1324–1313 1714–1710	773–769 553–549 1425–1421 842, 3461	1425–1421 773–769, 3461	773–769 1425–1421, 3461
PC2	Wavenumber (cm^−1^)	1691, 1336 1737, 518–511	1695–1691, 1066 838–804, 1027	1691, 518 1737, 804	1178–1170 1340, 1629–1621	1344–1340 1166, 730	1178–1170 1340, 1629–1621
PC3	Wavenumber (cm^−1^)	1726–1718 1186–1182 1232–1224	526–507 1232–1220 1683–1679	518–507 1224, 1683–1679	1425–1421 1309, 730	769, 410–403 3492–3488	1425–1421 1309, 730
**Calculation for Raman Data**							
PC1	Wavenumber (cm^−1^)	1622–1614 1583–1575, 735	1703–1695 1683–1680, 1155	1703–1695 1683–1680 1622–1618, 1579	1606–1595, 843 1286–1278	1610–1591 1286–1278, 843	1614–1595 846–843 1286–1278
PC2	Wavenumber (cm^−1^)	1683–1680 1699–1695 1174, 704	1699–1695 704–700, 1174	1699–1695, 1174 1618–1614	1174–1143 1602–1595 1286–1282	1170–1163 1506, 1159, 1255	1147–1124 1174–1163 850–846
PC3	Wavenumber (cm^−1^)	1699–1695 1174, 704–700	384, 1178–1174 735, 1618	1699–1695, 1174 704–700	684–681 1147–1120 1506–1502	1151–1143 684–681 1506–1502	683–681 1151–1143 1506–1502
